# Supplementary material for: Acceptability of fixed-dose combination treatments for hypertension in Kenya: A qualitative study using the Theoretical Framework of Acceptability
Source: PLOS Glob Public Health. 2025 Mar 18;5(3):e0003012. doi: 10.1371/journal.pgph.0003012 (PMC11918355; doi:10.1371/journal.pgph.0003012)
Supplement: S1 Text — (DOCX) [file pgph.0003012.s001.docx]

**HEALTH FACILITY ASSESMENT TOOL**

Information will be recorded using the checklist form below.

| Facility Code |  |
| --- | --- |
| Type of Facility |  |
| Date(s) of Visit |  |
| Researcher |  |

**Patient Flow**

| 1 | *Frequency and timing of patient visits (may include regularity, routine/specific clinics, opening & hours, operating days, whether there are specific NCD clinics)*  Also observe patient numbers, average waiting time, demographics at the clinics, time of attendance. |
| --- | --- |
|  |  |
| 2 | *Methods used to communicate with/contact patients in between clinic visits, if used (including contact details held)* |
|  |  |
|  | *Staff involved and role in process:* |
| 3 | *Procedure for prescribing medication for hypertension management (may include monitoring, consultation, issuing prescription, use of any decision support tools)* |
|  |  |
|  | *Staff involved and role in process:* |
|  |  |
| 4 | *Procedure for prescribing medication for secondary prevention of CVD (may include monitoring, consultation, issuing prescription, use of any decision support tools)* |
|  |  |
|  | *Staff involved and role in process:* |
|  |  |
| 5 | *Patient records (facility register, use of any tools)*  *What these include; paper or electronic, whether MOH NCD registers used- if so whether there is capacity to update it regularly.* |
|  |  |
|  | *Staff involved and role in process:* |
|  |  |

**Medicines**

| *1* | *Medicines currently prescribed /dispensed for hypertension*  For pharmacies and dispensaries, ask whether they currently have these in stock |
| --- | --- |
|  |  |
| *2* | *Procurement process for medication for hypertension management*  *Person responsible for ordering medical supplies at the facility?* |
|  |  |
| *3* | *Medicines currently prescribed /dispensed for secondary prevention of CVD* |
|  |  |
| *4* | *Procurement process for medication for secondary prevention of CVD* |
|  |  |
| *5* | *Procedure for medication dispensation at facility, including:* o *Opening hours* o *Payment procedures* o *Records* |
|  |  |
| *6* | *Procedure (informal/formal) if medicine is not available at facility, including:* o *Substitution*  o *Referral (formal/informal) elsewhere* o *Instruction to return*  *How long are the stockouts? Any regular pattern to stockouts? How they deal with stockouts e.g., redistribution.* |
|  |  |
| *7* | *Other facilities/services used by patients (health facilities, pharmacies, traditional medicine)* |
|  |  |

**Staff Roles**

List of staff involved at each stage of patient flow/medicines process, including:

- Job title
- Brief description of staff role in hypertensive/CVD patient management, including what happens if that staff member is unavailable.

| *Job Title* | *Description of role in role in hypertensive/CVD patient management:*   - *specific tasks* - *role sharing, procedure if individual is unavailable* |
| --- | --- |
|  |  |
|  |  |
|  |  |
|  |  |

**Additional Observations**

Record additional observations related to the study aim, for example advertisements for specific hypertension/CVD medication displayed in pharmacy. Observe patient-provider interaction/communication. Services provided and cost (service charter) Resources available at the facility (i.e., diagnostic equipment) Treatment guidelines/documents/charts etc.

|  |
| --- |

IMPLEMENT – CVD: Phase I

Topic Guide for Patients

| **Participant Study ID** | (Facility Code_P_00) |  |
| --- | --- | --- |
| **Interview Date** | (DDMMYY) |  |
| **Interview Completion** | 01= Completed  02=Partially Completed |  |
| **Interviewer** |  |  |

*First steps*

*1. Please introduce yourself to the participant and ask them to introduce themselves*

*2. Please ensure that* ***the participant has read (or has had read to them) the patient information sheet****, outlining the aims of the study, how their data will be used, and potential risks and benefits of participating, and* ***has signed the informed consent form*** *for participation.*

*3. Please confirm that the participant is happy to have their interview audio-recorded and that they know they can stop the interview at any point or refuse to answer any of the questions.*

*Once these steps are completed, you can proceed with the interview.*

*During the interview, you can use the questions below in the order that works best for your discussion with the patient. You can decide which questions to ask and when, based on the circumstances of the patient and what has already been covered during the interview. You can use the probes to explore themes in more depth, or to pick up on points raised earlier in the interview. It can be useful to ask participants for examples, and to use general probes for more depth such as ‘ could you tell me a bit more about x’.*

| **Age** |  |
| --- | --- |
| **Sex** |  |
| **Education Level** |  |
| **NHIF Coverage**  Ever enrolled Current status Package |  |
| **Travel to facility (mode, time, ease)** |  |

*Introduction: Thank you for meeting me. In this discussion, I would like us to talk about your experiences of managing blood pressure/heart disease, your views about your treatment/medications, and any challenges you have encountered. What we learn from your experiences will help in planning of treatment/medications for other patients with blood pressure/heart disease.*

1. **To start with, could you tell me about how you came to find out you have blood pressure, and when that was**?

*Probes:* *How did you feel after being diagnosed with blood pressure/heart disease? What came into your mind?*

2**) Separately from blood pressure, are you receiving healthcare/treatment for any other health conditions**? *[here it is up to the patient whether they want to offer further information about the conditions or not]*

If yes: Do you receive that care/treatment here [facility where recruited for study], or do you go to a different facility?

3) **Now I would like to talk about your experiences with treatment and medications for pressure. Where do you go for treatment and advice for blood pressure?**

*[Probe for any other sources as well as current facility, reasons for going to these, frequency of visits to sources mentioned]*

1. **Can you tell me about what happens during your consultation with the doctor?** [*Probes: How are decisions about treatment made? Does the doctor/pharmacist explain different options? Involve you in decisions/ask for your opinion? Does the doctor/pharmacist ask you about how you find the treatment prescribed?]*
2. **What medication(s) are you currently taking? Could you tell me about your experiences of taking this/these?**

*[Probes: Is there anyone who helps you with your medication?*

*Have you experienced any side effects taking any of your medications? If so what have you done? (have you carried on taking them, talked to Dr/pharmacist, stopped taking?)*

*Do you* ***sometimes miss some of your medication****? (why is that?)*

1. If you were to **explain to someone (e.g. friend, family) what each medicine does to help with your blood pressure/heart disease**, how would you describe that? [Reassure patient that’s fine if they don’t know/find it hard to explain]

*[Probes: for [X] medication, how would you describe what that does? For [y] medication, how would you describe what that does?]*

1. **Could you tell me about the process for you of getting and paying for blood pressure medication? How does it work?**

Who makes decisions about anti-hypertensive medications you use?

*[Probes: Do you need a prescription to get your medication?*

*Where do you get your medication from?*

*How do you decide where to get your medication from?*

[🡪 only if the patient describes having choice re source/type] *what is most important to you when you are getting medication?*

*[Probe for factors like who it is prescribed by, cost, availability, brand/generic/KEMSA supplied, packaging, recommendation of others, side effects]*

*How would you describe availability of medications?*  *How is the cost of medications?* [if affordability challenges are raised 🡪] *how do you manage these challenges? Any NHIF coverage/reimbursement?*

1. [depending on answers above**] Are you familiar with combination pills to treat blood pressure/heart disease?**

[**if yes**: can you tell me about your experiences with that? How do/would you feel about using it?]

Do you think there would be the benefits for you of using such kind of medication?

Do you have any concerns regarding the use of combination pills?

What kind of support would you need/require to make decisions regarding use of combination pills? [Link to response to ‘who makes decisions about anti-HT medications that you use?]

Should these medicines be available, would you be willing/open to using them? Why/why not? What would influence your decision to switch to combined medication?

[**if no**: There are some combination pills available in Kenya and other countries, which combine multiple existing blood pressure/heart disease drugs into one pill. If there was a combined pill available instead of several separate pills, how would you feel about using that?

*[Probe: What information or support would you need to make a decision about using the combination pill?]*

1. [if reported comorbidities] **Are you familiar with the use of combination pills for the treatment of other conditions?**

*[Probe: Experiences/ Impressions of using FDCs to treat these conditions - experiences of switching to FDCs, benefits/challenges using single molecules/FDCs?]*

1. **Thinking about blood pressure/ heart disease, if you were asked to describe it to someone who didn’t know about it, for example a neighbour who had just been told they had pressure and didn’t know what it was, how would you explain it?**

*[Probes: how would you describe causes of blood pressure/heart disease? How would you describe symptoms/ effects on the body? How would you describe any long-term impacts?]*

1. **Thinking about your own experiences, with pressure in general, how have you learnt about blood pressure?**

*[Probes: had you heard about blood pressure/heart disease before you were diagnosed?* ***Where*** *or* ***who*** *have you learnt about it from? family/friend with condition, healthcare providers, pharmacists, websites, information leaflets*]

If various sources: *probe re* ***which the patient prefers****/finds most useful and why (find* ***more reliable)***

1. **Could you tell me about anything you do** [yourself], **to look after yourself with blood pressure**  (manage this condition?)

*[Probes: Are there steps you have taken to manage this condition? (diet, exercise, medication?)]*

Have you experienced any challenges doing this?

1. **Could you tell me about anything that other people**, for example in your family, friends, church community, **do to help you look after yourself with blood pressure** in managing this condition?

*[Probes: In what ways? Does anyone help you to attend appointments, buy medications, remember to take medicines, encourage you? ]*

1. **Generally, how do you find your treatment and any adjustments you’ve made in your life, are working for you?**

*[Probes: Do you experience any symptoms of blood pressure/heart disease? Does your blood pressure/heart disease affect your daily life? If so, in what way(s)]*

1. **Is there anything else to do with treatment for pressure that you think we should be aware of?**
2. *Do you have any question?*

*Close and thank patient for their time. Reiterate how data will be used.*

**IMPLEMENT – CVD: Phase I**

**Topic Guide for Healthcare Providers and Pharmacists**

| **Participant Study ID** | (Facility Code_P_00) |  |
| --- | --- | --- |
| **Interview Date** | (DDMMYY) |  |
| **Interview Completion** | 01= Completed  02=Partially Completed |  |
| **Interviewer** |  |  |

*Introduction: Thank you for meeting me. In this discussion, I would like us to talk about your experience of treatment/ managing hypertension, challenges you/patients encounter in managing hypertension and your views about acceptability of different treatment options.*

1. **Can you describe your role as pertaining treatment of patients with HTN** *[Probe: How long have you been working in this role? What aspects of care are you responsible for pertaining to hypertension and CVD treatment/management?]*
2. **How would you describe the process of diagnosis and treatment of hypertension in this facility?**

What are the steps taken in screening, prescribing, dispensing, and monitoring of patients with hypertension**?** *[Probe: From talking to people, we understand that [summarize patient flow] could you correct me if I am misunderstanding? Are patients monitored after prescription? If so, how and how often? What happens to patients lost to follow up/defaulters?]*

**For Medical Officers/pharmacists:**

What guides your decisions when prescribing/dispensing medication? [*Probe: Process of establishing optimal regimens for individual patients? Who/where do you go for guidance if you have any questions/if you have uncertainties?]*

How would you describe the workload in prescribing/dispensing hypertension/CVD treatment? How does this compare to overall workload? Do you feel task-sharing could help address workload issues? If so, which cadres do you think would be able to step in?

**For Nurses:**

How would you feel about nurses sharing the role of prescribing for HTN with medical doctors when it comes to hypertension management? *[Probe: For which patient groups/ medication regime the patient is in? What should be the ideal?]*

**For Pharmacists/Nurses:**

Do you encounter any challenges with supply of hypertension/CVD medicine supply? If so, can you describe these?

[*Probe for medicine availability, procurement and storage, quality]*

How does your facility go around these challenges?

1. **Do you encounter any patient-related challenges in managing HTN/CVD treatment? If so can you describe these?**

How would you describe patients’ understanding of hypertension/CVD disease and its treatment?

Where do patients get information about hypertension disease and its treatment? *[Probe on patient’s knowledge before diagnosis and after diagnosis]*

Is there variation in knowledge among different patient groups? *[Probe: Age, gender, SES, time since diagnosis, family members with condition, patients with co-morbidities]*

1. **What do you think influences patients’ adherence to HTN medication?** *[Probe for availability, affordability, side effects, opinions of others in support network e.g., family, church,* concomitant use of traditional/ alternative medicines*?]*

What other challenges do patients report about medicine use?

Are there any particular groups of patients where there are more challenges with treatment?

What do you do to help patients overcome these challenges?

How do patients go around these challenges? *[Probe: About self-care and steps taken to practice self-management?]*

1. **Does your facility provide fixed dose combination treatment for hypertension?**
2. **If not providing:** Are you familiar with the concept of fixed-dose combination therapy? If not familiar: interviewer to explain. If aware: How did you learn about the FDC strategy? What do you think would be the advantages and disadvantages of using FDCs? Do you think FDC medications would improve HTN management and or patient experience? Is your facility planning to start providing FDC treatment? If yes, describe. If no, why?

**If providing:** What is your experience with FDC treatment for hypertension? When did you start providing FDC medications?

What kinds of patients are eligible for FDCs? What kinds of patients do you prescribe it to?

1. What kind of impact do you think it has on hypertension control? What about on patient experience?

Are there any challenges with use of FDC treatment? *[Probe on perception about FDCs, importance of dose titration, FDC medication availability]*

How would you suggest that these challenges could be addressed?

1. The 2018 Kenya National Guidelines for Cardiovascular Diseases Management produced by the ministry of health recommend the use of FDCs for hypertension. Are you aware of these guidelines? To what extent do you draw on these guidelines to prescribe anti-hypertensive for HTN patients? What’s your experience in practice in implementing these guidelines? [Probe: *Do you think there any challenges in implementing these guidelines?]* What’s your experience in relation to FDCs?
2. The ministry of health approved and included the following molecules of FDCs in Kenya’s EML *(lisinopril + hydrochlorothiazide; telmisartan + amlodipine; telmisartan + hydrochlorothiazide).*

For each combination, ask:

- Do you have experience in prescribing these?
- How do/would you feel about prescribing these molecules in combination pills?

[Probe: Is this commonly used? Would you have any concerns about prescribing these? Combinations that are more viable for Kenya?]

For Lisinopril+HCTZ: we are aware this was made available as ‘Zestoretic’ in some facilities through the HHA programme, subsidized by Astro Zeneca and supplied via MEDS. Did you have experience of prescribing this? How was your experience prescribing this? Patient experience? Do you know what happened to this programme? Are these combinations still in use? Why/why not?

1. How does (if used)/ might the use of FDC impact on the workload? Healthcare providers roles? *[Probe on acceptability by patients? Concerns by healthcare providers? Ethical considerations when interpreting the guidelines?]*

[A later phase of this study entails designing (with stakeholders) and evaluating a pilot intervention to improve implementation of FDCs for hypertension.]

1. What factors would you say support/ impede the implementation of FDCs in this county/country?

(Probe: At the system level, at the hospital level, at the prescriber level, at patient level? How can these factors be addressed?)

1. What support (information, material, financial) are available/ or would be needed to implement FDC for hypertension?

How sustainable do you think FDC treatment is? *[Probe: Are there any challenges with consistent supply?]*

Before we conclude, is anything else you would like to add on what we have discussed? *[Probe: do you think FDCs are a good treatment option and why/why not?]*
