# Supplementary material for: Acceptability of fixed-dose combination treatments for hypertension in Kenya: A qualitative study using the Theoretical Framework of Acceptability
Source: PLOS Glob Public Health. 2025 Mar 18;5(3):e0003012. doi: 10.1371/journal.pgph.0003012 (PMC11918355; doi:10.1371/journal.pgph.0003012)
Supplement: S2 Checklist — (DOCX) [file pgph.0003012.s003.docx]

Inclusivity in global research

PLOS’ policy on inclusivity in global research aims to improve transparency in the reporting of research performed outside of researchers’ own country or community and ensures that PLOS publications reporting global research adhere to high standards for research ethics and authorship. Authors of relevant research articles may be asked to complete the questionnaire below, which outlines ethical, cultural, and scientific considerations specific to inclusivity in global research. This questionnaire may be requested when researchers have travelled to a different country to conduct research, if research uses samples collected in another country, research with Indigenous populations or their lands, or if research is on cultural artefacts. Researchers travelling to another country solely to use laboratory equipment will not normally be required to complete the questionnaire. However, the questionnaire can be requested at the journal’s discretion for any submission – if you have been requested to complete this questionnaire by the PLOS journal you submitted to, please do so.

Please complete the questionnaire below and include this as a Supporting Information file with your manuscript. Note that if your paper is accepted for publication, this checklist will be published with your article in the supporting information files. Please ensure that you reference the checklist in the main body of your manuscript. We suggest adding a subsection ‘Inclusivity in global research’ to your Methods section and adding the following sentence: “Additional information regarding the ethical, cultural, and scientific considerations specific to inclusivity in global research is included in the Supporting Information (SX Checklist)”

The questions have been designed to be applicable to a wide range of study types, and there are subsections for both human subjects research and non-human subjects research. If any of the questions are not relevant to your research please mark them as “N/A” as appropriate.

**Ethical considerations, permits and authorship**

*This section is applicable to all research types.*

Provide details as to who granted permissions and/or consent for the study to take place in the Methods section of your manuscript. This should include the names of **all** ethics boards, governmental organizations, community leaders or other bodies that provided approval for the study. If individuals provided approval refer to these people by their role or title but do not list their name(s).

Reported on page number: 28

This study was approved by Kenya Medical Research Institute’s scientific and ethics review unit (SERU) (Approval NO: KEMRI/RES/7/3/1), National Commission of Science and Technology (NACOSTI) (Approval NO: NACOSTI/P/22/21524) and London School of Hygiene and Tropical Medicine Research Ethics Committee (28062). Permissions were also granted by the county government and each facility before commencement of the study. Written informed consent was obtained from all participants. Researchers took care to ensure that potential participants understood that participation was optional, and that participation or non-participation in the study would not affect the care which patients received.

If there were any deviations from the study protocol after approval was obtained please provide details of these changes in the Methods section of your manuscript.
Did this study involve local collaborators that are residents of the country where the research was conducted or members of the community studied? If you do not have any authors from said communities, please provide an explanation for this below.

Yes, Daniel Mbuthia (Co-Investigator and first author), Mary Gichagua, (Co-Investigator), Jacinta Nzinga (Co-investigator) and Peter Mugo (Co-investigator), and Ministry of Health officials from the counties involved in the project.

Reported on page number: n/a

Everyone listed as an author should meet PLOS’ criteria for authorship and all individuals who meet these criteria should be included in the author byline, rather than the acknowledgements. For further information please see the journal’s Authorship Policy.

**Human subjects research (e.g. health research, medical research, cross-cultural psychology)**

Did you obtain written informed consent from a representative of the local community or region before the research took place? How did you establish who speaks for the community? Details of written informed consent obtained from study participants should be reported separately in the Methods section of your manuscript.

The study received approvals from Kenya Medical Research Institute’s Scientific and Ethics Review Unit and the National Commission for Science, Technology and Innivation (NACOSTI) . We also obtained a written permission from the county government of Kiambu to conduct the research in the county.

Regarding informed consent, every study participant provided written informed consent as reported on page 11, Methods section.

How did members of the local community provide input on the aims of the research investigation, its methodology, and its anticipated outcome(s)?

Members of the local community (i.e. people using services at the study health facilities) did not provide input on the aims, methodology or anticipated outcomes of the research investigation. Local researchers including the Co-investigators PM, DM and MG (who is a Medical officer in the local health system), provided input on the aims, methodology, outcomes. However, members from the local community contributed to interpretation of findings in a Stakeholder Forum to discuss preliminary findings and identify priority themes for further exploration in subsequent analysis (Methods section, page 14).

When engaging with the local community, how did you ensure that the informed consent documents and other materials could be understood by local stakeholders?

Materials were designed by bilingual (English and Swahili) researchers based in Kenya, experienced in conducting research with local communities, following instutional guidance for study information sheets and consent documents. Materials were reviewed by the wider study team, which included collaborators experienced in providing health services, and by Kenya Medical Research Institute’s Communication and Consent Committee (CCC) as well as the Kenya Medical Research Institute’s Scientific and Ethics Review Unit. Materials were piloted and refined as needed.

During the informed consent process, the researcher took care to check the potential participant’s understanding of materials, and prompted for questions.

Will the findings of the research be made available in an understandable format to stakeholders in the community where the study was conducted (e.g. via a presentation, summary report, copies of publications, etc.)? Please provide details of how this will be achieved.

Findings from preliminary analysis of Round 1 interviews were presented in a Stakeholder workshop (26^th^ April 2023) to representatives from patient groups, community health promoters, health facility staff, county and national level stakeholders; feedback regarding areas to explore in greater depth informed subsequent analysis.

Targeted policy briefs summarising key findings and recommendations have also been developed and disseminated to the relevant stakeholders.

Findings from this study have also been shared with the wider research community at Kenya Medical Research Institute’s weekly seminar. Further, results from this study have also been presented in the Kenya Cardiac Society’s annual congress on 25^th^ July 2024.

**Non-human subjects research using specimens/ animals collected as part of the study, or those housed in archival collections. Examples include archaeology, paleontology, botany and zoology.**

Did the permission you obtained from a local authority to perform the study include an agreement on access to outputs and benefit sharing? This may include procedures to enable fair distribution of the benefits and resources arising from the research performed. Please include any details of Prior Informed Consent and Benefit Sharing Agreements obtained. These may be required by field-specific regulations, for example the Convention on Biological Diversity (CBD) and the associated Nagoya Protocol.

n/a – no non-human subjects research

If the material used in your study was imported, please A) provide the year it was imported and B) indicate whether permits were obtained to import/export the materials used, C) provide details of any permits obtained. If this information is not available, please indicate this.

n/a

If you used archival specimens, please state how the material used in your study was acquired by the institute it is held in and provide details of any permits obtained for the original excavations/ sample collection. If this information is not available, please indicate this.

n/a

How was the potential cultural significance of the materials collected in your study to local communities considered in your research design? Were Indigenous peoples and/or local researchers and institutions involved with archaeological excavations / collection of specimens? If so, please provide a description of their involvement.

n/a

If your manuscript includes photographs of human remains please indicate whether authors obtained permission from descendants or affiliated cultural communities to do so.

n/a
