# Supplementary material for: Acceptability of fixed-dose combination treatments for hypertension in Kenya: A qualitative study using the Theoretical Framework of Acceptability
Source: PLOS Glob Public Health. 2025 Mar 18;5(3):e0003012. doi: 10.1371/journal.pgph.0003012 (PMC11918355; doi:10.1371/journal.pgph.0003012)
Supplement: S1 Appendix — (DOCX) [file pgph.0003012.s004.docx]

Appendix 1

Theoretical Framework of Acceptability constructs applied to FDCs for hypertension.

| **Construct** | **Meaning** | **Meaning for acceptability of FDC treatment for hypertension for patients, caregivers, and healthcare workers** |
| --- | --- | --- |
| Affective attitude | How an individual feels about the intervention | How patients, caregivers and healthcare workers feel about FDC treatment |
| Burden | The perceived amount of effort that is required to participate in the intervention | The perceived amount of effort required for patients to use FDCs according to their prescribed use.  The perceived amount of effort required by caregivers to support patients to use FDC treatment  The perceived amount of effort required by healthcare workers to treat hypertensive patients using FDCs |
| Ethicality | The extent to which the intervention has good fit with an individual’s value system | The extent to which FDC treatment has a good fit with patients’/caregivers’/healthcare workers’ value systems |
| Intervention coherence | The extent to which the individual understands the intervention and how it works | The extent to which patients/caregivers understand FDC treatment and how it works *(i.e. understand the concept of combining separate hypertension medications in one pill, rather than understanding the mechanism of action of each medication*)  The extent to which healthcare workers understand FDC treatment and how it works |
| Opportunity costs | The extent to which benefits, values or profits must be given up to engage in the intervention | The extent to which benefits, values or profits must be given up:  - by patients to adhere to FDC treatment  - by caregivers to support patients to adhere to FDC treatment  - by healthcare workers to use FDCs to treat patients |
| Perceived effectiveness | The extent to which the intervention is perceived as likely to achieve its purpose | The extent to which patients/caregivers/healthcare workers perceive FDC treatment as likely to achieve management of hypertension |
| Self-efficacy | The participant’s confidence that they can perform the behaviour(s) required to participate in the intervention | Patient’s confidence that they can adhere to FDC treatment  Caregivers’ confidence that they can support patients to adhere to FDC treatment  Healthcare workers’ confidence that they can use FDCs to treat patients |
