# Supplementary material for: Acceptability of fixed-dose combination treatments for hypertension in Kenya: A qualitative study using the Theoretical Framework of Acceptability
Source: PLOS Glob Public Health. 2025 Mar 18;5(3):e0003012. doi: 10.1371/journal.pgph.0003012 (PMC11918355; doi:10.1371/journal.pgph.0003012)
Supplement: Attachment — Submitted filename: Responses to reviewers.docx [file pgph.0003012.s008.docx]

**Responses to reviewers**

| **Reviewer 1** | | |
| --- | --- | --- |
| **Comments** | **Responses** | |
| Abstract  “Poorly treated”? Or “poorly controlled”? | Thank you for the observation. We have edited to now read “poorly controlled”  We have also made a minor edit on the abstract on line 51 to read ‘knowledge about FDCs’ rather than ‘FDCs coherence’ to appeal to readers of the abstract who may not be familiar with the theoretical framework of acceptability. | |
| **Introduction**  The health system is not geared to dealing with this relatively new epidemic of NCDs. You should include information about the diagnosis of hypertension as the first step in controlling it – what proportion of the estimated cases are diagnosed? And then of those, what percept are on any treatment? And then of those, what percent are controlled? As with HIV, there’s a treatment cascade that starts with diagnosis | We have updated this section accordingly to show the treatment cascade and now it reads “In Kenya, for example, the prevalence of hypertension among people aged 18–69 years is estimated at 24.5%. However, only 15.6% -29.4% of these patients are diagnosed. Among those diagnosed, only 6.5% are on antihypertensive medication, and just 12.5% to 51.7% of those on medication have their blood pressure controlled. | |
| You should also consider adding provider training and confidence (or lack thereof) in treating hypertension. Check what’s in the literature on this topic and weave it into your ‘story’(this comes out clearly in your findings (lines 360-373) so you should update your introduction and discussion accordingly | Thank you. We have now updated this on the introduction and discussion section. | |
| Line 92 – What about the individual hypertension medications – are they available? How many of them? | Thank you for your comment. While individual hypertension medication are available in Kenya, the focus of our inquiry was on acceptability of fixed dose combinations for treatment of hypertension in Kenya. | |
| **Methods**  Line 157 – You found participants as patience in the clinic registers - had patients previously provided their consent to be contacted for purposes other than their clinical treatment? This should be mentioned here in addition to the general ethics statement required by the journal. | We have edited this section to show that patient recruitment was done prospectively from those attending clinic with the help of a triaging nurse, drawing on patients’ demographics in the registers to identify patients with ranging categories of experience of hypertension treatment.  The section now reads “On clinic days, with the help of a triaging nurse, the researchers prospectively and purposively selected patients living with hypertension that were attending the hypertension clinic, drawing on patient registers to identify patients representing a range of categories relevant to experiences of hypertension treatment.” | |
| Line 200 - Abductive thematic analysis – suggest defining “abductive” more completely as this is not widely used terminology | Thank you for noting this. In conjunction with addressing comments from Reviewer 2 we have revised this to ‘*a hybrid approach of inductive and deductive thematic analysis’*, which better describes the approach. | |
| **Results**  Line 212 – suggest clarifying – were all 24 patients known to be hypertensive? | All the patients were known to be hypertensive. As stated in the methods section in response to the comment above, we indicate that “the researchers prospectively and purposively selected **patients living with hypertension** that were attending the hypertension clinic” | |
| Was it typical to only have 1 FTC available? Is that all that’s typically ordered? | We observed that the only FDC commonly prescribed across the 4 facilities is a combination of losartan+ hydrochlorothiazide.  We clarify in the section “Ethicality: FDCs as cause of inequality in access to or affordability of treatment” that: “only combinations of losartan+ hydrochlorothiazide- less expensive than the individual drugs as separate pills- was the only FDC procured across the four health facilities despite not being on Kenya’s essential medicines list, supporting the idea that cost is an important consideration.” | |
| Were the 17 others prescribed anything at all? Or prescribed FTC (or something else?) and not taking? Or not prescribed at all? | Thank you for the comment. We clarify that “Of the 24 patients interviewed, seven were currently prescribed this FDC, while the rest were on separate pills” | |
| Line 245 – “limited awareness hampering understanding” – this is a repetitive phrase as awareness and understanding can be used interchangeably in this context. Push the concept a bit harder – consider looking into the field of “health literacy” and its effect on treatment adherence (for inclusion in your discussion section if relevant to your findings) | Thank you for noting this. This has now been renamed to “Intervention coherence: Low health literacy undermining intervention coherence” and we have expounded on it in the discussion section. | |
| Line 263 – Deferral of decision-making to healthcare workers – I think this is actually connected very closely with intervention coherence – it’s essentially the inverse, or the result of lack of treatment literacy. If you don’t understand it then you’ll just do what you’re told. | While these two concepts are closely connected, trust emerged as important construct that seemed to override intervention coherence. Patients expressed more confidence especially in healthcare workers in public health facilities as putting their (patients’) interest first over financial profit. We therefore interpreted this as a distinct construct of acceptability that goes beyond and above intervention coherence and we have expounded on this in the discussion section. | |
| Line 276-277 – this is your interpretation of the finding and belongs in your discussion section. Results are for reporting only the findings (no opinion or interpretation) | This has been removed | |
| **Discussion**  Lines 389-394 – following up my comment from Line 245 – this is a health literacy issue and there’s an entire field of literature to tap into, to further inform your background and discussion sections | We have edited the introduction and the discussion section accordingly. | |
| There’s an underlying implication to this paper that the problem is the drug combinations chosen – but that doesn’t take into account the health system challenges, from screening to drug selection at national level. Consider balancing the narrative a little more to reflect that FDCs are one aspect of controlling hypertension at the population level | Thank you for pointing this out. We have edited accordingly to recognize wider efforts to improve overall management of hypertension in Kenya. | |
| What about diagnosis of hypertension? How routinely is it screened for? | We have included in our discussion section a recommendation for a comprehensive approach in the management of hypertension including improving screening and diagnosis of patients, which should be done routinely and opportunistically in every patients’ clinical encounters, but also through active community screening and linkage to care through the community health promoters. | |
| If FDCs are already in the “essential medicines list” for Kenya, you need to narrow in on what the actual problem is – are they actually being ordered? Or are the monotherapy drugs being ordered? And if they are available, why aren’t they being used? | Thank you for your comment. This is indeed an important question and is one of the objectives of our broader study. We address this issue in a separate paper that explores health system factors that might affect implementation of FDCs. | |
| **Reviewer 2** | | |
| **Comments** | | **Response** |
| In line 40-45 include the name of the county to make it easier for reference and context without having to read the entire paper. | | Thank you for the observation. We have included the name of the county and now the section reads ‘We interviewed a total of 58 participants across four purposively selected health facilities in Kiambu county, in Kenya.’ |
| There is a bit of a mix up on the methodology used or rather the title is a bit of misleading given the methodology used in the study. The aim of the study is to explore the acceptability of the FDCs among patients, caregivers and health workers. In congruence with the title line 103-104 indicates that the conceptualization is based on the Theoretical Framework of Acceptability (TFA). In line 119-120 the study design indicates that the study relied on an explorative qualitative study design that allowed for exploration and understanding of phenomenon with limited prior evidence. In line 200 - 202 it is highlighted that the study used an abductive thematic analysis approach which builds on ‘grounded theory’ | | Thank you for noting that this was not clearly presented.  We have revised the text in lines 43-44 (abstract) and 200-205 (main text) to clarify our methodology. |
| Indicate the names of the specific hospitals that were included as part of the study within the study to provide proper context for the study. This makes it easier to have proper reference for the study and where specific study participants were selected from even though the county has been indicated. | | Thank you for your suggestion. We see why it would be useful for the readers to indicate specific names of hospitals that were included in the study. However, we have to balance that with respect to confidentiality. In some of the health facilities, it’s a small team of healthcare workers running the hypertension clinic and therefore revealing the names of these facilities would inadvertently compromise their confidentiality. |
| **Reviewer 3** | | |
| **Comment** | | **Response** |
| The manuscript is well written and it makes a good contribution to knowledge. However, the result presentation could be better with additional graphics and pictures for easy comprehension. The tables summarizing participant characteristics and facility details are helpful. However, additional visual aids (e.g., thematic maps or models) could be added to enhance the understanding of the findings. | | We have now included a diagram (figure 1) summarizing the key findings emerging from our data onto the constructs of the theoretical framework of acceptability. |
