# Supplementary material for: Acceptability of fixed-dose combination treatments for hypertension in Kenya: A qualitative study using the Theoretical Framework of Acceptability
Source: PLOS Glob Public Health. 2025 Mar 18;5(3):e0003012. doi: 10.1371/journal.pgph.0003012 (PMC11918355; doi:10.1371/journal.pgph.0003012)
Supplement: Attachment — Submitted filename: Review_FDCs hypertension Kenya.docx [file pgph.0003012.s005.docx]

Review: *Acceptability of fixed-dose combination treatments for hypertension in Kenya: a qualitative study using the Theoretical Framework of Acceptability*

Abstract

- “Poorly treated”? Or “poorly controlled”?

Introduction

- The health system is not geared to dealing with this relatively new epidemic of NCDs. You should include information about the diagnosis of hypertension as the first step in controlling it – what proportion of the estimated cases are diagnosed? And then of those, what percept are on any treatment? And then of those, what percent are controlled? As with HIV, there’s a treatment cascade that starts with diagnosis
- You should also consider adding provider training and confidence (or lack thereof) in treating hypertension. Check what’s in the literature on this topic and weave it into your ‘story’(this comes out clearly in your findings (lines 360-373) so you should update your introduction and discussion accordingly
- Line 92 – What about the individual hypertension medications – are they available? How many of them?

Methods

- Line 157 – You found participants as patience in the clinic registers - had patients previously provided their consent to be contacted for purposes other than their clinical treatment? This should be mentioned here in addition to the general ethics statement required by the journal.
- Line 200 - Abductive thematic analysis – suggest defining “abductive” more completely as this is not widely used terminology

Results

- Line 212 – suggest clarifying – were all 24 patients known to be hypertensive?
- Was it typical to only have 1 FTC available? Is that all that’s typically ordered?
- Were the 17 others prescribed anything at all? Or prescribed FTC (or something else?) and not taking? Or not prescribed at all?
- Line 245 – “limited awareness hampering understanding” – this is a repetitive phrase as awareness and understanding can be used interchangeably in this context. Push the concept a bit harder – consider looking into the field of “health literacy” and its effect on treatment adherence (for inclusion in your discussion section if relevant to your findings)
- Line 263 – Deferral of decision-making to healthcare workers – I think this is actually connected very closely with intervention coherence – it’s essentially the inverse, or the result of lack of treatment literacy. If you don’t understand it then you’ll just do what you’re told.
- Line 276-277 – this is your interpretation of the finding and belongs in your discussion section. Results are for reporting only the findings (no opinion or interpretation)

Discussion

- Lines 389-394 – following up my comment from Line 245 – this is a health literacy issue and there’s an entire field of literature to tap into, to further inform your background and discussion sections
- There’s an underlying implication to this paper that the problem is the drug combinations chosen – but that doesn’t take into account the health system challenges, from screening to drug selection at national level. Consider balancing the narrative a little more to reflect that FDCs are one aspect of controlling hypertension at the population level
- What about diagnosis of hypertension? How routinely is it screened for?
- If FDCs are already in the “essential medicines list” for Kenya, you need to narrow in on what the actual problem is – are they actually being ordered? Or are the monotherapy drugs being ordered? And if they are available, why aren’t they being used?
