# Supplementary material for: Acceptability of fixed-dose combination treatments for hypertension in Kenya: A qualitative study using the Theoretical Framework of Acceptability
Source: PLOS Glob Public Health. 2025 Mar 18;5(3):e0003012. doi: 10.1371/journal.pgph.0003012 (PMC11918355; doi:10.1371/journal.pgph.0003012)
Supplement: Attachment — Submitted filename: critique on FDC study ph.docx [file pgph.0003012.s006.docx]

**Review report**

The study by Mbuthia et al. focused on the acceptability of fixed-dose combination (FDC) treatments for hypertension in Kenya using a specific theoretical framework (Theoretical Framework of Acceptability).

Abstract

The abstract is well written with a good overview of the study's background, methods, results, and conclusions.

Background

The background is well-written and establishes the significance of hypertension as a global health issue, particularly in LMICs. The rationale for using FDCs is well-explained, as are the specific challenges in the Kenyan context.

Methods

The methods section is generally well described with justified study design and detailed data collection process.

Results

Presentation of Findings: The results are presented clearly, with appropriate categorization according to the TFA constructs.

Illustrations: The tables summarizing participant characteristics and facility details are helpful. However, additional visual aids **(e.g., thematic maps or models) could be added to enhance the understanding of the findings.**

Discussion

The discussion appropriately interprets the findings

Conclusion

The conclusion succinctly summarizes the study's main findings and their implications for hypertension treatment in Kenya.

Overall, the manuscript provides good insights into the acceptability of FDCs for hypertension in Kenya, with practical implications for policy and practice. However, **additional visual aids in the results section could improve clarity of the findings.**
